# Machine Learning Application of Transcranial Motor-Evoked Potential to Predict Positive Functional Outcomes of Patients

**DOI:** 10.1155/2022/2801663

**Published:** 2022-05-20

**Authors:** Mohd Redzuan Jamaludin, Khin Wee Lai, Joon Huang Chuah, Muhammad Afiq Zaki, Khairunnisa Hasikin, Nasrul Anuar Abd Razak, Samiappan Dhanalakshmi, Lim Beng Saw, Xiang Wu

**Affiliations:** ^1^Department of Biomedical Engineering, Faculty of Engineering, Universiti Malaya, Kuala Lumpur 50603, Malaysia; ^2^Department of Electrical Engineering, Faculty of Engineering, Universiti Malaya, Kuala Lumpur 50603, Malaysia; ^3^Center of Environmental Health and Safety, Faculty of Health Sciences, Puncak Alam Campus, Universiti Teknologi Mara Selangor, Bandar Puncak Alam 42300, Selangor Darul Ehsan, Malaysia; ^4^Department of Electronics and Communication Engineering, College of Engineering and Technology, Faculty of Engineering and Technology, SRM Institute of Science and Technology, SRM Nagar, Kattankulathur, Chengalpattu, Chennai, Tamil Nadu, India; ^5^Department of Orthopaedic Surgery, Sunway Medical Centre, Selangor, Malaysia; ^6^School of Medical Information & Engineering, Xuzhou Medical University, Xuzhou 221000, China

## Abstract

Intraoperative neuromonitoring (IONM) has been used to help monitor the integrity of the nervous system during spine surgery. Transcranial motor-evoked potential (TcMEP) has been used lately for lower lumbar surgery to prevent nerve root injuries and also to predict positive functional outcomes of patients. There were a number of studies that proved that the TcMEP signal's improvement is significant towards positive functional outcomes of patients. In this paper, we explored the possibilities of using a machine learning approach to TcMEP signal to predict positive functional outcomes of patients. With 55 patients who underwent various types of lumbar surgeries, the data were divided into 70 : 30 and 80 : 20 ratios for training and testing of the machine learning models. The highest sensitivity and specificity were achieved by Fine KNN of 80 : 20 ratio with 87.5% and 33.33%, respectively. In the meantime, we also tested the existing improvement criteria presented in the literature, and 50% of TcMEP improvement criteria achieved 83.33% sensitivity and 75% specificity. But the rigidness of this threshold method proved unreliable in this study when different datasets were used as the sensitivity and specificity dropped. The proposed method by using machine learning has more room to advance with a larger dataset and various signals' features to choose from.

## 1. Introduction

Disc herniation and prolapsed disc that compresses the nerve roots in the lumbar region can cause sensory and motor disturbances, which contribute to low back pain, leg pain, and weakened leg's motor strength [1]. Decompression surgery or discectomy is a treatment surgery of removing the bulging disc from compressing the nerve roots.

The use of intraoperative neuromonitoring (IONM) in the lumbar discectomy procedure helps to monitor the integrity of the nervous system from further injury. IONM modalities such as somatosensory-evoked potential (SSEP), motor-evoked potential (MEP), and electromyogram (EMG) are commonly used in lumbar surgery. However, SSEP has limited function since it only monitors specific nerve roots that are innervated from the S1 level when the posterior tibial nerve is stimulated [2]. It is also less sensitive to the changes of nerve root function because the signal is the result of a summation of neural signal from multiple segments before it enters the spinal cord [3]. EMG can show continuous nerve root events since it is free running, but it has a high false positive rate and low specificity in determining events that are significant for the surgeon to alert on [4]. MEP or transcranial motor-evoked potential (TcMEP) has high sensitivity and specificity towards nerve root injury, but it relies on the alarm criteria used and which myotome is monitored [5].

Besides showing potential nerve injury during surgery, several studies which were reviewed by [6] presented in Table 1 had shown that improvement to the TcMEP signal is significant towards positive functional outcomes of patients. Most of the researches utilized amplitude increment as the improvement indicator. However, they have no common agreement on what is the increment percentage that is considered significant to show actual improvement in the postsurgery patient outcome. This paper aims to use machine learning algorithm to classify TcMEP signals into no improvement and improvement.

## 2. Related Works

### 2.1. Literature Reviews on Automated IONM, Objective Interpretation on IONM, and Machine Learning Applications on IONM

Currently, automated feature in commercial IONM machines only exists for triggered EMG (trigEMG) modality. It is done by attaching/clipping a stimulating device with a conductive surgical instrument. While the conductive surgical instrument is advanced deeper inside the spine body, increasing continuous stimulus is applied automatically to detect any nearby nerve structure so that nerve injury can be avoided [16]. The technique is straightforward, such that if the nerve is nearby the instrument, less stimulus is required to trigger the nerve. If a higher stimulus is required to trigger the nerve, it means that the nerve is further away from the instrument and safe to operate. It uses a basic principle by setting a threshold level and does not require an algorithm to interpret the signal. However, this technique in Malaysia is often neglected because of the high additional cost incurred by the IONM service. Moreover, this modality does not serve as a prognostic tool to determine the neurological condition of the patient.

There were several researches that used IONM to predict patients' functionality outcome by using an automatic algorithm. The first research was done by [17] to apply a deep learning algorithm on visual evoked potential (VEP) modality in order to detect changes to the VEP signal during sellar region tumours surgery. Another research was done for automatic SSEP interpretation by [18] to be used in cardiac surgery for peripheral nerve injury prevention. The application of deep learning and automatic interpretation of VEP signal and SSEP signal is supported by the fact that there is a universal acceptable criterion of normal and abnormal VEP and SSEP signal patterns to make the prediction possible. As far as our knowledge is concerned, no TcMEP signal has been applied for automatic patients' functionality outcomes categorization.

The interpretation of TcMEP is more difficult even by the IONM team because of high variability trial-to-trial, anaesthetic effect, and high sensitivity [19]. Interpretation of TcMEP signal drop or usually known as alarm criteria is based on the complexity of the surgical procedure [20]. Furthermore, if changes are observed on TcMEP signals by the monitoring personnel, he or she has to go through a checklist of troubleshooting before the final interpretation is made to minimize the possibility of false positive or false negative events [21, 22]. Among the checklist that needs to be clarified before the surgical reversal is initiated are technical aspects (electrodes and machine connections and stimulation parameters) and anaesthesia/systemic (patient's mean arterial pressure (MAP), blood pressure, body temperature, and anaesthesia used).

### 2.2. Literature Review on the Applicability of TcMEP as Prognostic Tool to Justify Positive Functional Outcome of Surgery

In creating a meaningful prognostic application to justify the outcome of the surgery, we first need to identify if TcMEP has been used for that particular purpose in the literature. Considerable efforts have been exerted to address the ability of TcMEP to predict the risk of injuries to the nervous system, but only a few have focused on proving the ability of TcMEP to predict positive functional outcome of the patients intraoperatively. Studies that showed TcMEP as a prognostic tool to correlate the TcMEP improvement with the improvement after surgery were reviewed by [6] and are presented in Table 1.

A previous study by Barley et al. [7] utilized TcMEP on a 15-year-old boy who presented with upper and lower limbs motor weakness during a tethered spinal cord release procedure. Only the right abductor pollicis brevis (APB) of the upper limb was obtained, and no other responses were observed. Postdetethering revealed that left APB response appeared and increment of right APB amplitude, but only left upper extremity had notified improvement after surgery. Another research had proven that patients with improved TcMEP signal had better American Spinal Injury Association Impairment Scale (AIS) [13]. He et al. [15] presented a case report of a patient that had percutaneous endoscopic lumbar discectomy with TcMEP monitored and discovered that the TcMEP amplitude increment after the decompression was associated with low back and leg pain relief immediately after the patient was awake. Another study used IONM on 12375 patients who had spinal surgeries over 25 years with 386 patients exhibiting IONM signals improvement [10]. However, there were several IONM modalities (including TcMEP) that they monitored without specifying the improvement indicator. One patient had a permanent neurological deficit despite having IONM signal improvement, but this is statistically significant with the 14 true negative cases. Meanwhile, Wang et al. [12] had found that improvement of TcMEP signals (specifically amplitude rather than latency) in 59 patients who went through cervical laminoplasty or laminectomy was highly correlated with a modified Japanese Orthopaedic Association scale or mJOA improvement rate. Even though these studies had proven that the improvement on TcMEP had a high correlation with functional patient outcome, these five studies did not mention any specific improvement criteria from the IONM signal that they used to indicate significance towards postsurgery improvement.

Prospective research was done by Piasecki et al. [2] on patients who went through lumbar decompression surgery with TcMEP monitored. By using 20% of TcMEP area under the curve (AUC) increment as improvement criteria, they compared the findings with the Zurich Claudication Questionnaire (ZCQ) assessment of patients. It was found that the patients with improved TcMEP's AUC had higher ZCQ scores. A study conducted by Rodrigues et al. [9] had TcMEP monitored during a decompression surgery of a 22-year-old male athlete who was having lumbar pain and weakness on the right foot. The patient had a 30% of TcMEP amplitude increased after the discectomy procedure and was able to return to competitive athletic activities a month after surgery. Visser et al. [11] had used 200% of TcMEP amplitude increment criteria as significant to show actual patients' neurological improvement. This threshold was suggested by them to rule out any influence possibilities on the TcMEP generations. They found out that the MEP improvement should also be associated with the symptom's duration of less than 6 months for it to be significant. Another research that was done by Voulgaris et al. [8] compared the TcMEP outcomes with pain visual analogue scale (VAS) in patients who underwent lumbar decompressive laminectomy. The TcMEP improvement criteria were set on a 50% increment mark, and it was shown that the patients who had more than 50% TcMEP-increased amplitude had better VAS score at 12-month follow-up compared to the others who had lower amplitude increment. Wi et al. [14] found out that patients with more than 100% amplitude increment had better Motor Index Scoring System (MISS). It was then concluded by Wi et al. [14] that improvement of IONM signals could indicate the success of decompression. However, among these five studies, they have no common amplitude increment percentage that they used in their studies to indicate significance (>50% in [8] and >200% in [11]).

The proposed approach of this paper requires features that are the signal characteristics or parameters that we need to feed to the machine learning models. Hence, we will make use of the TcMEP signal's parameters that were already established as presented in Table 1, which are the peak-to-peak amplitude and the AUC values. We also added the onset latency of the signal as one of the features selected for the machine learning models. The onset latency has not been used to predict the functional positive outcome. But it was used in [23] as the alarm criteria to indicate significant postoperative motor deficit. It was proven in the study that the onset latency had high sensitivity and specificity (100% and 84%, resp.) towards the detection of motor deficit compared to the amplitude threshold criteria (using more than 70% drop of amplitude as a significant indicator) at 100% sensitivity and 72% specificity. But when they combined both criteria (amplitude and onset latency), the sensitivity remained at 100%, and the specificity increased to 93%. Hence, in our study, we decided to include the onset latency parameter to be experimented as one of the features to run with the machine learning models.

In this paper, we are proposing a machine learning approach to be applied to the TcMEP signal that could identify patients that would have positive functional outcome and patients that has no changes from presurgery to postsurgery. We will also compare the efficacy of our results with two of the presented approaches in Table 1 from [8, 11] that utilized the TcMEP amplitudes threshold (>50% of amplitude increment and >200% of amplitude increment, resp.) as their improvement criteria since we are also using the peak-to-peak amplitude as one of the features that we used for the machine learning.

## 3. Methods

### 3.1. Data Source

The TcMEP data are the selected 55 patients who underwent lumbar disc decompression surgeries and patients who underwent instrumentation and correction surgeries from August 2021 until January 2022 at Sunway Medical Centre, Malaysia. Among the 55 patients, 13 patients had presurgery motor weakness and developed positive motor improvement after surgery (named as group MI for motor improvement), 34 patients had presurgery symptoms such as numbness, back pain, and leg pain which had improved after surgery (named as group PNR for pain and numbness relief), and eight patients had no symptoms before surgery and after surgery (named as group NC for no changes). The actual outcomes of the postsurgeries were recorded based on the attending surgeons' evaluations. The eight patients who had no symptoms before surgery and after surgery were patients who only had instrumentation and correction surgeries.

All of the patients went through the surgery with the aid of IONM consisting of SSEP, MEP, and EMG modalities as requested by the surgeons. The myotomes involved in all of the surgeries were different depending on the spine levels that were being operated on. Since all of the operations involved spine lumbar L2 and below, the monitoring included vastus lateralis (VL) muscle (innervated from L2 to L4 nerve roots), tibialis anterior (TA) that innervated from the L5 nerve root, and abductor hallucis (AH) that innervated from S1 to S2 nerve root. Reference electrode was placed on hand muscle abductor digiti minimi (ADM) or abductor pollicis brevis (APB), whichever is easily accessible. Only TcMEP data were chosen for this study. Since the parameters used on the IONM and the approaches of the IONM setup on the patients were not experimental, they were all applied accordingly to the necessities of the surgeries and the surgeons' requests, and this study is not categorized as a prospective study. This study was approved by Sunway Medical Research Council, and since it was only a retrospective study, no informed consent was obtained from the patients.

### 3.2. Intraoperative TcMEP Monitoring

TcMEP monitoring was applied using the NIM Eclipse E4 system (Medtronic, Minneapolis, MN). The TcMEP was stimulated by using corkscrew electrodes placed at the C3 and C4 over the motor cortex on the scalp according to the International 10–20 System scalp electrode placements. The monitoring electrodes were placed at different muscles bilaterally by using dual subdermal needle electrodes.

The stimulus intensity varied from 250 V to 600 V. Train pulse stimulus of three to five pulses was applied to most of the patients to overcome the response variability or inconsistency. Sometimes, double train stimulation of five pulses and three pulses was used if it was difficult to elicit TcMEP response. Interstimulus interval was set to be either at 5 ms or 10 ms, which was based on which produced the better MEP response. Overall, the ideal TcMEP stimulation would be to elicit a response of more than 20 *µ*V of peak-to-peak amplitude for each channel with minimal patient movement.

Short-acting muscle relaxant was used in all of the patients during intubation, and the anaesthetic protocol was maintained with total intravenous or TIVA for the rest of the surgery. However, some of the anaesthetists applied inhalational agents such as desflurane and/or sevoflurane during intubation which made the baseline reading establishment difficult and affected the interpretation of IONM. One anaesthetist had used midazolam on the patient.

Ideally, the baseline reading was obtained after the patient was intubated and before any incision was made, but most of the time, the muscle relaxant used during intubation lasted longer and required some time to completely wear off. So, the baseline readings were at least obtained before the spine area was fully exposed and operated on. The TcMEP was then stimulated from time to time to compare with the baseline reading so that any deterioration could be detected and reversed if needed.

### 3.3. Feature Selection and Machine Learning

**Figure 1:**
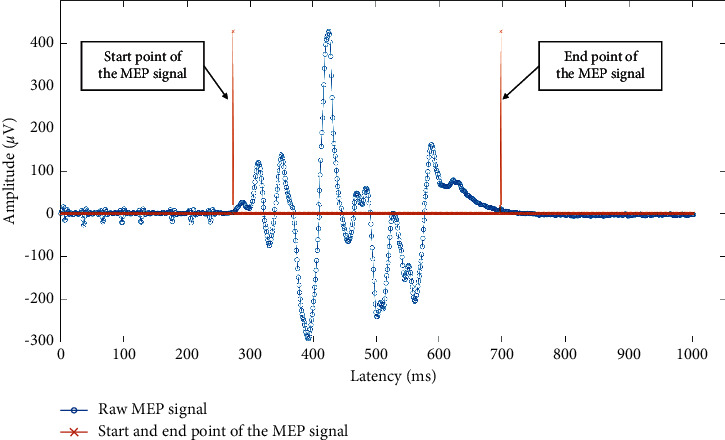
Start and end point marks at the raw MEP signal.

**Figure 2:**
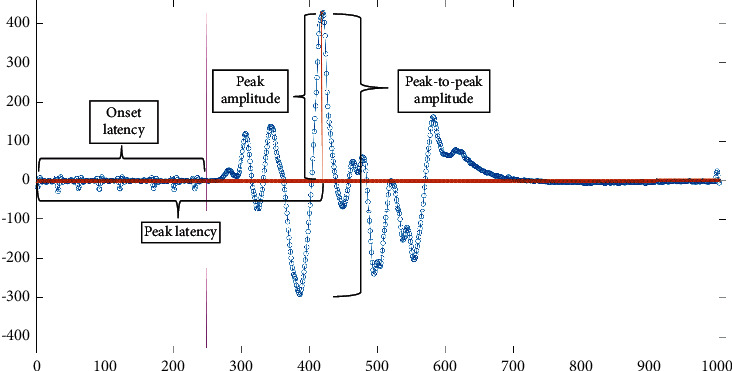
Onset latency, peak-to-peak amplitude, peak amplitude, and peak latency labels on raw MEP signal.

The features that are used for the proposed approach in this paper were the onset latency which is the start point of the MEP signal in ms [23], the peak-to-peak amplitude in *µ*V, and AUC.  Figure 1 shows the frame of an MEP signal from the start of the response to the end of the response.  Figure 2 shows the definition of onset latency, peak latency, peak amplitude, and peak-to-peak amplitude of an MEP signal. These values were obtained straight from the NIM Eclipse E4 system. The AUC was obtained through NIM Eclipse E4 by selecting the section of TcMEP response which is within the start point of the signal until the end point of the signal as presented in Figure 1.

We went through the recorded patients' history in the IONM machine to identify which of the patients had presurgery neurological symptoms and which of the patients had no presurgery neurological symptoms. These data were obtained from preclinical evaluation made by the surgeons on their patients and were recorded in NIM Eclipse E4 during the surgery for future references. Then, we further investigated along the timeline of each patient's comment history to find at what time was the best TcMEP baseline achieved and the final reading for analysis. Then, we recorded three TcMEP features (peak-to-peak amplitude, onset latency, and AUC) from these two times. The readings were obtained from one target muscle and three reference muscles. The target muscle is the muscle that indicates weakness or pain. The three reference muscles were supposed to represent the asymptomatic myotome of the patient and not involved in the surgical site such as the hand muscles or the side of the limb that was not symptomatic.

The relevance behind this was because it is the similar approach used by the IONM technicians intraoperatively, which is that any change (either drop or increase of amplitude or latency delay) of a certain myotome, especially the target myotome, is compared with reference readings from reference myotome. This is based on the idea that there should be no significant changes in the reference myotome during the surgery, and any changes to the target myotome can be interpreted as significant and require further attention.

The final features that were used for the machine learning were obtained from the percentage difference between features from the baseline reading against features from the final reading. We also added the averaged values of peak-to-peak amplitude, onset latency, and AUC from all of the four muscles as another feature.

Consequently, the 55 patients were split into training and testing at two different ratios, which were 70 : 30 (39 patients for training and 16 patients for testing) and 80 : 20 (44 patients for training and 11 patients for testing). Since the data was quite small, we carefully selected the patients from MI, PNR, and NC groups for them to have equal numbers in both training and testing. The baseline readings of three MI patients and one NC patient were highly influenced by anaesthesia. The anaesthetist for these four patients used midazolam during the intubation, which highly suppressed the baseline reading. We included these samples because firstly we need the NC sample because the sample number of NC is limited and small. Secondly, if the proposed approach is able to classify the different groups despite the baseline readings being poor because of the anaesthetic factor, this study could be beneficial for current and future works since the chance of getting a false positive result is high with these types of samples. The two ratios (70 : 30 and 80 : 20) were chosen as it is commonly accepted as the training and testing ratio in machine learning studies. Moreover, through our experimental procedures, the results obtained from the ratios 70 : 30 and 80 : 20 are worth presented and significant in this study compared to the results obtained from the other ratio sizes.

Test run was done with different sets of features:

- Set A: all features included.
- Set B: only target and three reference muscles peak-to-peak amplitudes and onset latencies.
- Set C: target and three reference muscles peak-to-peak amplitudes, onset latencies, and AUCs.

We utilized the classification learner application in MATLAB to run the samples for training. After the training run was done with all of the models available in the classification learner application, we identified which of the models had 100% accuracy. We then chose these models with 100% of accuracy to run testing with the remaining samples. Specific models that were used and the results achieved will be presented in Section 4.

We also utilized the improvement criteria presented by [8, 11], which were more than 50% of peak-to-peak amplitude increment and more than 200% of peak-to-peak amplitude increment, respectively, to compare the accuracy of their proposed threshold against our proposed model. The way we applied these criteria was by setting the rule as follows:

- We used the target muscle for each patient. Target muscle is the muscle that is claimed to be having symptoms before surgery.
- We obtained the percentage difference between the amplitudes from the baseline reading and the final reading of the target muscle's TcMEP response.
- For 50% rule, if the difference in amplitude is more than 50%, then the result will be a true improvement. Otherwise, the result will be no changes.
- For 200% rule, if the difference in amplitude is more than 200%, then the result will be a true improvement. Otherwise, the result will be no changes.

## 4. Results

The results are shown in Table 2 for 70 : 30 ratio of training and testing samples and Table 3 for 80 : 20 ratio of training and testing samples. The first two columns of both tables showed the results of using the 50% amplitude increment criteria and 200% amplitude increment criteria on the same testing samples for comparison with the proposed method. The second column of each table represents Set A, the third column of each table represents Set B, and the fourth column of each table represents Set C as described in Section 3.3. Only the machine learning models that achieved 100% accuracy during the training session were selected for the testing session, and these are presented in both tables.

The machine learning models' ability to detect the positive outcome showed high sensitivity percentage. In Table 2, Fine KNN and Weighted KNN achieved 100% sensitivity in Sets A, B, and C. But they had no specificity, which means they were unable to identify patients who have no positive outcome. However, 50% amplitude increment criteria showed high specificity percentage on the same test samples used, which was 75% and had a high sensitivity of 83.33%.

Meanwhile, in Table 3, Fine KNN in Set B and Set C achieved 100% and 87.5% sensitivities, respectively, with 33.33% specificity in both sets. This indicates that the additional feature, which was the AUC in the Fine KNN model, dropped the sensitivity percentage. But when the 50% amplitude increment criterion was applied to the dataset in Table 3, the sensitivity and specificity dropped to 75% and 67%, respectively, compared to the result achieved by using the dataset in Table 2.

On the other hand, the other machine learning models that achieved 100% accuracy during the training session (Weighted KNN, Ensemble Subspace KNN, and Ensemble Bagged Trees) in Sets A, B, and C produced 0% specificity in both Tables 2 and 3. Ensemble Subspace KNN in Table 2 had 8.33% false negative and 12.5% false negative in Table 3 when AUC was added as one of the features compared to when only peak-to-peak amplitude and onset latency were used. In fact, in Table 3, when peak-to-peak amplitude, onset latency, and AUC were used as features, Fine KNN, Ensemble Bagged Trees, and Ensemble Subspace KNN exhibited a 12.5% false negative rate. There was no false negative rate at both tables when Set B was used as the features.

The 200% amplitude increment criteria had lower sensitivity in both tables, which is 25% but had relatively higher specificity (75% in 70 : 30 ratio and 67% in 80 : 20 ratio) than the machine learning approach.

Overall, the proposed machine learning approach had higher sensitivity and lower specificity compared to the improvement criteria proposed by [8, 11]. We will look in-depth on what are the possibilities that lead to these findings in Section 5.

## 5. Discussion

In this paper, we proposed the utilization of machine learning to analyse the characteristics of TcMEP signals in order to group them objectively into TcMEP signals that significantly show positive functional outcome or TcMEP signals that do not indicate any positive functional outcome. We also compared the performance of the proposed approach with the amplitude criteria presented in the literature, and we found that the proposed method could potentially assist in the interpretation of TcMEP monitoring during lower lumbar decompression surgeries.

Among the tested machine learning models, Fine KNN with peak-to-peak amplitude and onset latency as the features achieved high sensitivity (100%) on TcMEP response to predict whether there will be a positive functional outcome to the patient or not. Several limitations to the proposed method are acknowledged. The proposed method needs input from the IONM technical personnel to select the best baseline signal for comparison with the final signal. Some of the surgeries finished in under two hours of time, for which the baseline readings were still influenced by muscle relaxant and inhalational agents induced during intubation, causing some false positive events to the analysis. After several encounters of difficulty to achieve the best baseline in some of the patients who had the same anaesthetist, the anaesthetist was enquired about his/her technique, and he/she admitted that midazolam was used in all of his/her patients. It is understood from the previous study made by [24] that midazolam could also highly suppress the TcMEP response. These patients were still included in this study for the sake of testing whether the prediction can still be made even though anaesthetic influence was involved.

With the small number of patients from the NC group, the machine learning was unable to distinguish clearly between patients who were expected to have improvement and patients who were expected to maintain their strength and functionality. This caused the low specificity in most of the models even though Fine KNN in 80 : 20 ratio exhibited 33.33% specificity. Additional numbers of data in the NC group would help in increasing the prediction capability of the proposed approach. Moreover, it can be observed that the specificity of Fine KNN improved when more samples were added to the training dataset (70 : 30 ratio compared with the 80 : 20 ratio). But we did not go for 90 : 10 ratio as 10% of samples for testing are too little and do not produce a significant finding.

Another finding was when AUC was added as the feature (in Set A and Set C), the false negative rate increased for Fine KNN, Ensemble Bagged Trees, and Ensemble Subspace KNN. Even though the study made by [2] presented that they used 20% AUC increment as the criteria of improvement, AUC had no significance in increasing the accuracy of the machine learning models in our study when it was added to the models' feature.

The NC group introduced by this paper should not be assumed as patients who develop postsurgery deficit. Although the introduction of a group of patients who develop postsurgery deficit would be another valuable information that could lead to better patient management intraoperatively, we have not come across enough data for training and testing purposes (only two cases appeared during the period of the data collection).

**Figure 3:**
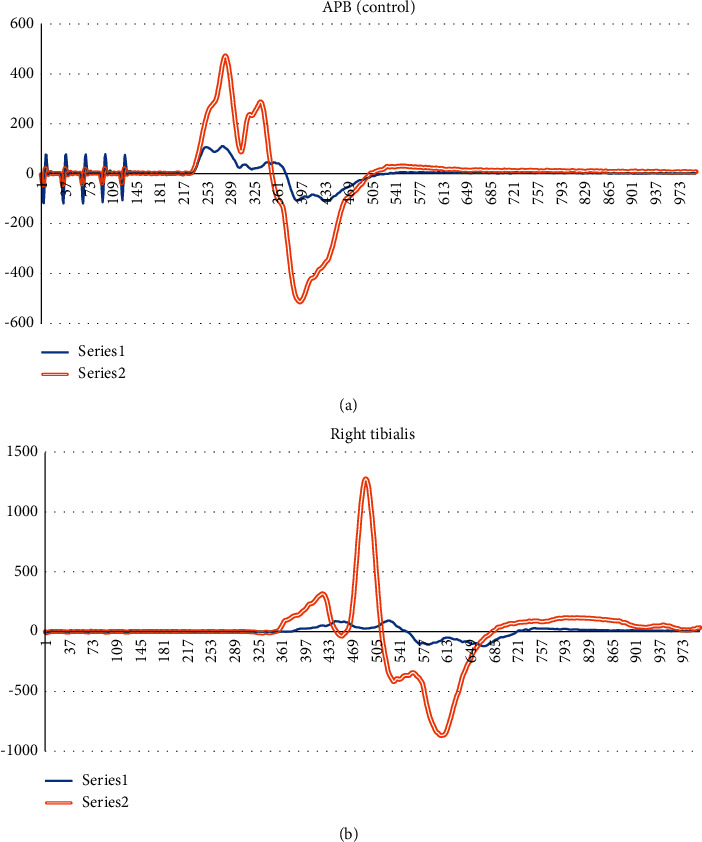
TcMEP responses of an NC sample. (a) Control muscle APB. (b) Target muscle right tibialis. Series 1 in both figures are baseline readings, and series 2 in both figures are final readings.

Surprisingly, the 50% amplitude increment criteria achieved relatively high sensitivity and high specificity. It proves that this criterion is still relevant in most cases if it is added with additional input and interpretation from the neuromonitoring technician or specialist. However, it was observed with different sample sets (test samples in Table 2) that the sensitivity and the specificity by using this criterion dropped. Moreover, this threshold method could only be applied onto one target muscle. If we look further into one sample that this method predicted wrongly on one NC sample, the target muscle was set as right tibialis while the control muscle was set as the APB. This sample is shown in Figure 3. The behaviour of this sample shows that the baseline readings were influenced by muscle relaxants from the intubation period since the final response of the APB muscle (nonsurgical site) increased drastically compared to the baseline response. By using this technique, the sample can be mistakenly identified as improved.

Meanwhile, the 200% criterion had a low true positive rate because the threshold was too high, and most of the MI and PNR samples were unable to achieve this criterion. Hence, we suggest that the threshold method is inflexible in such a way that if the threshold is too high, the rate of true positive will be lower, and if the threshold is too low, most samples will be predicted as having improvement, thus increasing the false positive rate.

We propose for future works that any prediction system by using the TcMEP signal can incorporate with the input from the comment history that records the events during the surgery. The system should be able to screen through the comment history to look for certain keywords such as “baseline,” “after decompression,” or “final TcMEP” so that the system can locate the best TcMEP signal for the interpretation process automatically.

## 6. Conclusion

IONM has been used widely to prevent or reduce the risk of nerve injury in spine surgeries. Alarm criteria to predict significant nerve injuries have been widely studied and established. However, the study of the use of IONM to predict improvement in patient's symptoms can be improved further. Predicting the positive outcome of the decompressive surgery on the lower lumbar level intraoperative via IONM will be valuable information for the surgeon to make a decision on the depth of decompression needed. If the decompression is not done enough, patient might not get the intended result, but if the decompression is done too deep and too long, the risk of nerve injury increases, and it will prolong the duration of the surgery.

In this paper, we utilized machine learning models to predict the outcome of the intraoperative TcMEP. The best machine learning model that achieved a high percentage of sensitivity and specificity percentages was Fine KNN with only peak-to-peak amplitude of the target muscle and three reference muscles used as the feature parameters. The Fine KNN model performance was able to predict a positive functional outcome from the TcMEP response with high sensitivity but with low specificity. With the limitations on the whole TcMEP modality itself, any drop or change to the signal requires human technical involvement before the actual interpretation is passed. Thus, it is advised that the proposed system can only act as an assistant to the monitoring personnel. On the other hand, we managed to show that the 50% amplitude increment criterion is relevant as a predictor of positive functional outcome.

## Figures and Tables

**Table 1:** The summary of studies that indicated that TcMEP can be used as a prognostic tool [6].

Number	Reference	Number of samples	IONM modalities used	Stimulation parameters	Muscles used to monitor MEP	Improvement criteria	Results
1	Barley et al. [7]	One (15-month-old boy)	TcMEP and SSEP	C1-C2 scalp electrode positioning, current stimulation (145 mA to 187 mA for the left extremities and 175 mA to 200 mA for the right extremities)	Bilateral quadriceps femoris, tibialis anterior, gastrocnemius, sphincter, abductor pollicis brevis, and abductor hallucis	Not mentioned	TcMEP response of the left APB had an increment in amplitude. The patient had observable left upper extremity improvement
2	Voulgaris et al. [8]	25 (2 had no IONM results)	TcMEP and EMG	C1-C2 with multipulse current stimulation, 0 mA to 200 mA, stimulus duration 0.2 ms to 0.5 ms	Not mentioned	>50% MEP amplitude improvement	17 patients with >50% improvement had better VAS score improvement
3	Rodrigues et al. [9]	One (case report)	SSEP, MEP, and free running EMG	C3-C4 stimulation	Not mentioned muscles' names specifically but monitoring covered L3-S2 myotomes	Not mentioned	MEP improved as much as 30%, and patient had returned to sports
4	Raynor et al. [10]	386 patients had IOM signals improvement out of 12375 patients who had spinal surgeries over 25 years	DNEP, TcMEP, spontaneous EMG, triggered EMG, and dermatomal SSEP	C3-C4 TcMEP scalp electrode stimulation montage	Upper extremity TcMEP was recorded from deltoid, flexor/extensor carpi radialis, and/or abductor digiti minimi/abductor pollicis brevis. Lower extremity TcMEP was recorded from anterior tibialis, medial gastrocnemius, and/or extensor hallucis longus	Not mentioned	The results did not mention specifically TcMEP improvement, but out of the modalities used, 88.7% of patients had IOM signals improvement, but one patient out of this percentage had permanent neurological deficit
5	Visser et al. [11]	74 patients	TcMEP	Cz-Fz with monophasic stimulation and C3-C4 with biphasic stimulation	For the lower limbs, the quadriceps muscle (L2-L4), the tibialis anterior muscle (L4-L5), the hamstrings (L5-S1), or the gastrocnemius muscle (S1–S2). For cervical, the bilateral trapezoid muscle (C2–C4), the biceps (C5–C6), and triceps muscle (C7–C8) of the arm; the extensor muscles of the forearm (C6–C7); or the abductor digitus V muscle (C6–C8)	>200% of amplitude increment	There is a correlation between the duration of symptoms onset and the MEP improvement. MEP improvement can be accurate if the symptoms' onset duration is less than half a year
6	Wang et al. [12]	59 patients who had cervical myelopathy who underwent laminoplasty or laminectomy	MEP and SSEP	Not mentioned	Not mentioned	Not mentioned	Patients who had MEP signals improvement had a significant mJOA improvement rate. MEP amplitude was found to be a more accurate parameter compared to MEP latency in predicting surgery outcome
7	Dhall et al. [13]	32	EMG, MEP, and SSEP (not used for the study)	100 V–1000 V constant voltage stimulation, C1-C2 anodal stimulation, double train with a total of 9 pulses, 50 ms pulse width, 1.7 ms interstimulus, and 13.1 ms ISI	Not mentioned	Comparison with AIS grade and BASIC score of MRI images	MEP outcome (present) highly correlated with better AIS grade and BASIC grade
8	Piasecki et al. [2]	18	MEP and SSEP (not used for the study)	50 V–150 V C1-C2 biphasic stimulation, 5 to 7 train pulses, 500 Hz, and 1 ms interstimulus pulse	One upper limb muscle (control), bilateral tibialis anterior/bilateral abductor hallucis	>20% of AUC MEP; > 50% of ZCQ score	The MEP improvement was related to the early follow-up functional outcome
9	Wi et al. (2019) [14]	29 patients who had improvement in IONM signals out of 317 cases	MEP and SSEP	Not mentioned	Upper extremity TcMEP was recorded from deltoid, triceps, and thenar muscles. Lower extremity TcMEP was recorded from anterior tibialis and abductor halluces	Comparison with MISS, SF-36, JOA, NDI, and Oswestry Disability Index	The patients with MEP improvement had a better MISS improvement rate, while the patients with SSEP improvement only had a better SF-36 improvement rate
10	He et al. [15]	One (case report)	MEP and free running EMG	Not mentioned	Bilateral iliopsoas, rectus femoris, tibialis anterior, and medial gastrocnemius	Not mentioned	MEP improvement aligned with the patient's relieved symptoms

**Table 2:** Prediction performance of the models with 70 : 30 training samples and test samples ratio as the ability to identify the positive outcome.

			All features				Target and reference p2p amplitude and onset latency			Target and reference p2p amplitude, onset latency, and AUC			
	>200% method	>50% method	Fine KNN	Weighted KNN	Ensemble Subspace KNN	Fine KNN	Weighted KNN	Ensemble Bagged Trees	Ensemble Subspace KNN	Fine KNN	Weighted KNN	Ensemble Bagged Trees	Ensemble Subspace KNN
True positive	25.00%	83.33%	100.00%	100.00%	100.00%	100.00%	100.00%	100.00%	100.00%	100.00%	100.00%	100.00%	91.67%
True negative	75.00%	75.00%	0.00%	0.00%	0.00%	0.00%	0.00%	0.00%	0.00%	0.00%	0.00%	0.00%	0.00%
False positive	25.00%	25.00%	100.00%	100.00%	100.00%	100.00%	100.00%	100.00%	100.00%	100.00%	100.00%	100.00%	100.00%
False negative	75.00%	16.67%	0.00%	0.00%	0.00%	0.00%	0.00%	0.00%	0.00%	0.00%	0.00%	0.00%	8.33%

**Table 3:** Prediction performance of the models with 80 : 20 training samples and test samples ratio as the ability to identify the positive outcome.

			%All features				%Target and reference p2p amplitude and onset latency					%Target and reference p2p amplitude, onset latency, and AUC			
	>200% method	>50% method	Fine KNN	Weighted KNN	Ensemble Bagged Trees	Ensemble Subspace KNN	SVM Fine Gaussian	Fine KNN	Weighted KNN	Ensemble Bagged Trees	Ensemble Subspace KNN	Fine KNN	Weighted KNN	Ensemble Bagged Trees	Ensemble Subspace KNN
True positive	25%	75%	75.00%	100.00%	100.00%	87.50%	100.00%	100.00%	100.00%	100.00%	100.00%	87.50%	100.00%	87.50%	87.50%
True negative	67%	67%	33.33%	0.00%	0.00%	0.00%	0.00%	33.33%	0.00%	0.00%	0.00%	33.33%	0.00%	0.00%	0.00%
False positive	33%	33%	66.67%	100.00%	100.00%	100.00%	100.00%	66.67%	100.00%	100.00%	100.00%	66.67%	100.00%	100.00%	100.00%
False negative	75%	25%	25.00%	0.00%	0.00%	12.50%	0.00%	0.00%	0.00%	0.00%	0.00%	12.50%	0.00%	12.50%	12.50%

## Data Availability

All the data are available from the list of references. Data presented in Figure 1, Figure 2, Figure 3, Table 2 and Table 3 were collected from Sunway Medical Centre, Malaysia upon medical ethics approval.
